# Virtual reality exposure therapy in panic disorder: a pilot study

**DOI:** 10.1192/bjo.2021.672

**Published:** 2021-06-18

**Authors:** Clara Gitahy Falcão Faria, Veruska Andrea Santos, Marcos Fidry Muniz, Mariana Costa do Cabo, Antonio Egidio Nardi, Rafael Chistophe da Rocha Freire

**Affiliations:** 1 Institute of Psychiatry, Federal University of Rio de Janeiro; 2 Institute of Psychiatry, Federal University of Rio de Janeiro, Full Professor of Psychiatry, Federal University of Rio de Janeiro; 3 Associate professor, Queen`s University

## Abstract

**Aims:**

To ascertain if virtual reality exposure therapy (VRET) is an effective add-on tool in the treatment of Panic Disorder (PD).

**Background:**

The exposure to virtual stimuli has been studied as a useful treatment for PD. However, the studies with PD are still scarce and use dissimilar protocols, with effectiveness varying according to the protocol applied.

**Method:**

Eight PD patients received VRET as an add-on treatment to pharmacotherapy. The treatment protocol consisted of eight sessions. The first session is for the patient to understand the treatment and to answer the questionnaires. The second and third sessions were to prepare the patients for exposures with breathing training using diaphragmatic breathing and others breathing techniques to manage anxiety. From the fourth to eighth sessions, the patients followed a hierarchy of tasks during virtual reality exposure. Clinicians rated the Clinical Global Impression Scale (CGI) and the Panic Disorder Severity Scale (PDSS). The patients rated the Diagnostic Symptom Questionnaire (DSQ); the Mobility Inventory (MI), the Anxiety Sensibility Index (ASI-R), the Beck Depression Inventory (BDI), the Beck Anxiety Inventory (BAI) and the WHOQOL-BREF before and after the protocol. After all exposures, the Igroup Presence Questionnaire (IPQ) was applied to measure the sense of presence experienced in the virtual environment. The virtual environment simulated the subway of Rio de Janeiro.

**Result:**

There were no statistically significant improvements in the CGI-S, PDSS, BAI, MI or WHOQOL. There was a significant improvement in the BDI scores (P = 0.033). There was a trend towards improvement of anxiety measured by the ASI-R (P = 0.084) and of panic symptoms measured by the DSQ (P = 0.081) scores. There was also a significant improvement of sense of presence (IPQ – general presence) through the exposure sessions.

**Conclusion:**

Our study demonstrated that VRET as an add-on to pharmacological therapy could benefit PD patients. Despite the lack of significant differences in the means, the dispersion of PDSS and BAI scores were smaller after treatment compared to before treatment, suggesting that patients with more severe anxiety, panic and agoraphobia symptoms benefited more of the VRET protocol so, at the end of the treatment, differences were found in important measures of panic. Randomized controlled clinical trials are warranted to confirm the efficacy of VRET.

This study was funded by the Brazilian National Council for Scientific Development (Cnpq). The authors report no conflicts of interest.

